# Crosstalk Between Mesenchymal Stromal Cells and Tumor-Associated Macrophages in Gastric Cancer

**DOI:** 10.3389/fonc.2020.571516

**Published:** 2020-10-09

**Authors:** Ping Zheng, Wei Li

**Affiliations:** ^1^Department of Laboratory Medicine, The First People’s Hospital of Lianyungang, Lianyungang, China; ^2^Center of Research Laboratory, The First People’s Hospital of Lianyungang, Lianyungang, China

**Keywords:** mesenchymal stromal cells, tumor-associated macrophages, gastric cancer, tumor microenvironment, extracellular vesicles

## Abstract

Tumor microenvironment (TME) consisting of distinct cell types including stromal cells and immune cells has recently emerged as a pivotal player in tumor development and progression. Mesenchymal stromal cells (MSCs) and tumor-associated macrophages (TAMs) are two representative cells in the TME with plastic properties. This review will focus on the evolution of phenotypes and functions of either MSCs or TAMs, which is “educated” by the TME, as well as interactions between MSCs and TAMs contributing to the distinct stages of tumor biology in gastric cancer. MSCs exert immunoregulatory effects on macrophages and polarize them toward M2-like TAMs, via cell–cell contact and paracrine or extracellular vesicle (EV) transfer mechanism. In turn, M2-TAMs modulate the transition of “naive” MSCs into tumor-derived MSCs, which possess a more potent pro-tumor role than the parent. Moreover, the cross talk between MSCs and TAMs could contribute to cancer biology by inducing the EMT process, metastasis, immune invasion, and immunotherapy resistance in cancer cells. However, molecular mechanisms underlying interactions between MSCs and TAMs in gastric cancer progression need to be thoroughly elucidated, which may provide attractive targets for making promising novel strategies for gastric cancer therapy.

## Introduction

Gastric cancer is the fourth most commonly diagnosed malignant tumor worldwide and considered as the third leading cause of cancer-related death, following lung and liver cancer (1). Despite a drop in the mortality rate due to the advancements of pathological diagnostic programs and therapeutic approaches, gastric cancer remains a prevalent disease with poor prognosis (2, 3). Therefore, continuous attempts are urgently needed to discover new and effective targets for the clinical therapy of gastric cancer. With the advancements in cancer research at present, tumor microenvironment (TME) has risen as a prominent promising issue for developing the therapeutic strategies (4, 5) in cancer treatment.

As the microenvironment where tumor cells exist in, TME is composed of blood vessels, lymph vessels, stromal cells (such as fibroblast, pericytes, and adipocytes), immune/inflammatory cells (such as lymphocytes and macrophages), extracellular matrix (ECM), secreted proteins, RNA, and small organelles (4). Extensive researches have revealed the critical role of TME in tumor initiation, progression, metastasis, recurrence, and drug resistance (6, 7). Moreover, bidirectional interactions between the distinct components of TME have been proved to regulate many aspects of cancer biology (8). Among the cells in gastric cancer TME, mesenchymal stromal cells (MSCs) and tumor-associated macrophages (TAMs) are two predominant elements, and their cross talk has been shown to immensely contribute to the development and progression of tumor growth and metastasis (9, 10).

As non-hematopoietic stromal cells with the capacities to self-renew and differentiate into fibroblast, adipocyte, and osteoblast lineages, MSCs are recruited from bone marrow and incorporated into the TME to promote tumor growth and metastasis (11). Accounting for approximately 0.01% of the cells in solid tumor tissue, MSCs have been identified to have pro-tumor functions by stimulating tumor cell proliferation, supporting the growth of cancer stem cells and promoting the EMT process of tumor cells (12, 13). In particular, MSCs play an important role in the immunoregulation of immune cells (such as TAMs and neutrophils) resulting in the generation of an immunosuppressive microenvironment (14). In turn, the phenotypes and functions of MSCs can also be regulated by immune cells within the TME (15).

As reported, epithelial–mesenchymal transition (EMT) is associated with various tumor functions, including tumor initiation, tumor stemness, tumor cell migration and invasion, and resistance to anticancer drugs (16, 17). The process of EMT represents a conversion of an epithelial cell into an elongated mesenchymal cell. The mechanisms of EMT involve loss of cell junction and polarity, disintegration of cytokeratin filaments and desmosomes, and migration and invasion of the newly formed cells with a mesenchymal phenotype (18, 19). Given the important pro-tumor roles of either MSCs or TAMs in gastric cancer, the cross talk between MSCs and TAMs may contribute to tumor growth or metastasis through the promotion of EMT process in gastric cancer epithelial cells.

In this review, we discuss the evolution of phenotypes and functions of either MSCs or TAMs in gastric cancer and the emerging roles of interactions between MSCs and TAMs in the pro-tumor progression and clinical immunotherapy of gastric cancer.

## Methodology

For this review, a MEDLINE PubMed database was used for searching the related papers. Our database searching included the following terms: “mesenchymal stromal cells,” “tumor-associated macrophages,” “gastric cancer,” “polarization,” and “transition.” Criteria of inclusion: all relevant studies on the interactions between mesenchymal stromal cells and tumor-associated macrophages, and the contribution of their cross talk to gastric cancer progression, were considered for analysis in our review. Criteria of exclusion: information for the editorials, letters to publishers, and low-quality articles were excluded from the analysis. Based on the criteria of inclusion/exclusion, the titles, abstracts, and full-text articles in English language were analyzed and concluded.

## The Pro- and Antitumor Properties of MSCs

Due to their tumor tropism and immunosuppressive properties, MSCs have been proved to exhibit diverse biological functions in tumor (20). Numerous studies have provided the evidence that tumor-derived MSCs could promote the growth and metastasis of a variety of malignances, via the secretion of trophic factors or cell-to-cell contact with the other TME cells (21, 22). MSCs emerging in these studies were obtained from tumor tissues by the method of enzymatic digestion and identified based on the morphological, phenotypic, and differentiated parameters of the isolated cells. It was reported that MSCs isolated from the primary ovarian tissue could enhance the proliferation, colony formation, and tumorigenesis of cancer cells by secreting high levels of IL-6 (23). Cervical cancer-derived MSCs were reported to play an important role in suppressing the antitumor immune response in cervical cancer through the purinergic pathway (24). In gastric cancer, tumor tissue-derived MSCs could prompt tumor growth and metastasis through the secretion of IL-8 (25).

Conversely, other literatures reported that MSCs resident in the TME could diminish tumor development and progression, which generates a contradictory role of MSCs. In an experiment on breast cancer, MSCs were demonstrated to suppress cancer cell growth and sensitize the cancer cells to radiotherapy through inhibiting the STAT3 signaling pathway (26). Moreover, MSCs and their conditioned media also have a major role in anti-proliferation of ovarian cancer cells and can be considered as a potential therapeutic tool in ovarian cancer (27).

To explain this controversy of MSCs’ role in cancer biology, various evidences have emerged such as the heterogeneity of MSC preparations, the age or health of the MSC donor, or the experimental model (28, 29). In 2010, a novel methodology was developed and established to induce the conventional mixed pool of MSCs into two distinct uniforms, termed as MSC1 and MSC2 (30). Similar to macrophage polarization into M1 (classic) and M2 (alternative) subtypes, the authors demonstrated that MSCs could be induced into MSC1 and MSC2, which were found to have divergent effects on cancer growth and spread *in vitro* and *in vivo*. Primarily, MSC1 had an antitumor effect, whereas MSC2 could promote tumor growth and metastasis, which is similar to the conventional MSCs (31). In addition, coculture of the distinct phenotypes of MSCs with cancer cells also reflected differences in the secreted bioactive factors in a contact-dependent or independent mode. Thus, this original paradigm for MSCs has provided a new understanding of the contradictory role of MSCs. However, the introduction of MSCs into the subtype of MSC1 or MSC2 within the TME, particularly by the other TME cells, has not been elucidated.

## The Categories and Roles of Tams in Gastric Cancer

As a key component of cancer-associated inflammation, macrophages have been confirmed to impact many hallmarks of cancer (32, 33). Within the tumor beds, macrophages can be categorized into the primitive “embryonic-derived tissue macrophages” and the “bone marrow-derived recruited macrophages,” which arise from the peripheral blood monocytes and are attracted into the TME by chemokines (34). Due to the heterogeneity of TAMs, the inherent plasticity in their biology and phenotype classification suggests a complex role of macrophages in the distinct stages of cancer (35).

Commonly, tumor macrophages are broadly classified as either M1 (classic) or M2 (alternative) activated subset. During different stages of cancer biology, the specific phenotypes of activated macrophages are determined by the signals or stimuli from the surrounding TME (36). A combination of interferon-γ (IFN-γ) and lipopolysaccharide (LPS) or granulocyte-macrophage colony-stimulating factor (GM-CSF) could polarize the macrophages into an M1 phenotype, which is pro-inflammatory and mediates antitumor immunity. In addition to the expressions of iNOS, IDO, and MHC II, M1 macrophages are characterized by the specific cytokine profile including IL-1, IL-6, IL-12, and TNF-α, which brings about a Th1 response. Conversely, M2 macrophages are polarized by IL-4 and IL-13 or colony-stimulating factor 1 (CSF1), which can be further classified into subsets of M2a, M2b, M2c, and M2d. Characterized by the high expressions of arginase-1, macrophage scavenger receptor 1 (CD204), and mannose receptor (CD206), M2 macrophages produce high levels of IL-10, vascular endothelial growth factor (VEGF), and matrix metalloproteinase, which can contribute to an anti-inflammatory response and pro-tumorigenic functions, such as tumor growth, survival and angiogenesis.

Although the polarized states of macrophages are dictated by cues from the TME, TAMs have been demonstrated to possess an M2-like phenotype prominently in tumor tissues (37, 38). In gastric cancer, various studies have presented the critical roles of M2 TAMs in the initiation, progression, and metastasis (39). It can affect the outcomes of therapy and the overall prognosis of gastric cancer patients through diverse mechanisms (40). High density of CD163^+^ (CD206^–^) TAMs with concurrent high CD68 expression was reported to be associated with upregulated immune signals and improved patient survival in gastric cancer (41). It was also demonstrated that CD163^+^ TAMs were involved in promoting the peritoneal dissemination of gastric cancer via IL-6 secretion (42). In addition, gastric cancer-derived exosomes have been shown to induce the generation of PD1^+^ TAMs with M2 phenotypic and functional characteristics, which could produce a large amount of IL-10, impair CD8^+^ T-cell function, and thereby create conditions prompting cancer progression (43). Therefore, M2 TAMs have emerged as a promising effective target for gastric cancer treatments. Nevertheless, knowledge gaps in better understanding of the generation and functions of TAMs still exist.

## MSC-Mediated Polarization of Tams Contributes to Gastric Cancer Progression

**FIGURE 1:**
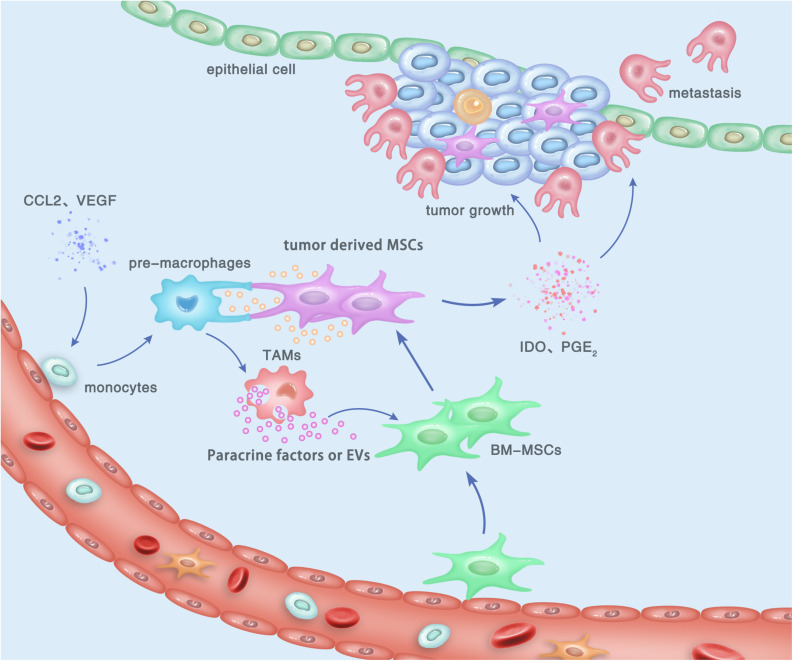
Cross talk between tumor-derived MSCs and TAMs, and its contribution to the development and progression of gastric cancer within the TME. By cell-to-cell contact, paracrine effect, or EV transfer, tumor-derived MSCs induce the polarization of macrophages to obtain an M2-like phenotype, which is termed as TAMs and can promote the proliferation, invasion, and metastasis of gastric cancer cells. In turn, M2 TAMs can trigger the transition of naïve BM-MSCs into tumor-derived MSCs, which also play a tumor-supportive role in gastric cancer.

MSCs have drawn researchers’ attention due to their immunoregulatory ability. Accumulating evidences suggested that MSCs possess a broad spectrum of immunoregulatory properties, including both the innate and adaptive immunity (44, 45). MSCs can regulate the infiltration, proliferation, differentiation, maturation, and functions of immune cells including leukocytes, dendritic cells (DCs), granulocytes, and monocyte/macrophages (46, 47). MSC-derived extracellular vesicles (EVs) were shown to specifically modulate CD4^+^ active T-cells into a regulatory profile by miRNA and metabolism shifting (48). In addition, MSCs were reported to suppress the maturation and migration of DCs and so that they could serve immunoregulatory ability by modulating the Ag-presenting function of DCs (49). By secreting multiple cytokines and growth factors, macrophages were demonstrated to play a key role in both advancing and resolving inflammatory diseases such as cancer, wound healing, and autoimmune diseases (50, 51). The regulating effect of MSCs on the phenotypes and functions of macrophages has drawn much attention, since it may be involved in distinct stages of gastric cancer biology (52, 53). Generally, the communication between MSCs and TAMs can be performed by the following pathway: (a) direct cell-to-cell contact, mediated by cell surface molecules; (b) indirect contact via the secreted active molecules; and (c) indirect contact by cell-induced EVs such as exosomes (Figure 1).

### Immunoregulatory Effect of MSCs on TAMs via Cell-to-Cell Contact

By direct cell-cell contact, MSCs can modulate the shift of phenotypes and functions of macrophages resident in the TME of several solid tumors. MSCs have been proved to trigger an M2 anti-inflammatory phenotype in macrophages with high secreting levels of IL-10 and TNF-α and low levels of IL-6 and nitric oxide via cell-to-cell contact (54). In addition, the phagocytosis of apoptotic thymocytes was also enhanced in MSC-treated macrophages. After coculturing with monocytes by direct contact, follicular lymphoma-derived MSCs (FL-MSCs) were demonstrated to play a key role in the recruitment and polarization of monocytes into TAM-like cells (55). By cell sorting, the cocultured macrophages were picked up and showed a strong inhibition of TNF secretion together with increased releases of pro-angiogenic and pro-tumoral factors, including IL-10, IL-6, and VEGFA. However, the mechanism for immunoregulatory effect of MSCs on TAMs is complicated and various in distinct stages of cancer biology, and hitherto there has been no report concerning the modulation of MSCs on M2 polarization of macrophages via cell-to-cell contact in gastric cancer. Although MSCs have the capacity to home to tumor beds from the earliest stage of cancer, they still have poor survival ability and transitory persistence within the TME of tumor tissue (56). Thus, paracrine effect by secretion of soluble factors may contribute predominantly to the MSCs’ immunoregulatory effect on the fate of macrophages in gastric cancer.

### Immunoregulatory Effect of MSCs on TAMs via Paracrine

Paracrine is a type of indirect cell-cell communication by which a cell can alter the behavior of the adjacent cells by transforming the active molecules or factors (57). MSC-secreted TGF-β could skew LPS-simulated macrophage polarization into an M2-like phenotype, reduce the inflammatory reactions, and improve the phagocytic ability via the Akt/FoxO1 pathway (58). It was also demonstrated that MSC-induced polarization of macrophages did not depend on direct cell-to-cell contact. Precondition of MSCs highly strengthened their potential to promote the IL-6-dependent M2b polarization of macrophages (59). In another study, conditioned medium from MSCs was shown to triggere the M2 macrophage polarization in the TME of gastric cancer, which in turn promoted the invasion and migration of gastric cancer cells by inducing the process of EMT (60). Therefore, MSCs play a pivotal role in directing the phenotypes and functions of TAMs toward an M2-like subset by secreting the paracrine factors such as TGF-β and IL-6, which subsequently orchestrates a pro-tumor niche. However, the mechanism for MSCs’ modulating effect on TAMs’ polarization by paracrine still needs to be thoroughly illuminated for searching the clinical therapeutic targets of gastric cancer.

### Immunoregulatory Effect of MSCs on TAMs by EVs Transfer

Recently, increasing attention has been drawn on the EVs as another non-contact modality for mediating the immunoregulatory effect of MSCs on TAMs. Composed of small particles including exosomes and microvesicles, EVs are an important agent of intercellular communication by transferring proteins or nucleotides from donor cells into recipient cells (61). Exosomes from human or mouse tumor-educated MSCs were shown to drive accelerated breast cancer progression by inducing differentiation of monocytic myeloid-derived suppressor cells into highly immunosuppressive M2 TAMs at tumor sites (47). In an experiment on non-small lung cancer, hypoxia-pre-challenged MSC-secreted EVs induced the M2 polarization of macrophages via downregulation of PTEN partly by miR-21-5p delivery (62). In another report, the release of EVs was also adopted by MSCs as a modulator in inducing the polarization of TAMs. Internalization of MSC-EVs by TAMs significantly elicited their switch from M1 to M2 phenotype, conformed by a downregulation of the M1 marker Nos2 and an increased expression of the putative M2 markers Arg1, Ym1, and CD206 (63). However, verification on the role of MSC-EVs in regulating the phenotypes and functions of TAMs has not been reported in gastric cancer, which is urgently needed to be performed in the future.

## TAM-Triggered Transition of MSCs in Gastric Cancer

As mentioned above, the properties of TAMs at tumor sites can be promptly regulated by tumor-infiltrated MSCs. In turn, the differentiation or transition of MSCs may also be affected by TAMs within the TME. It was shown that “naive” MSCs originating from the bone marrow could be attracted into the tumor tissues and “educated” by the inflammatory microenvironment into a tumor-associated phenotype (64). These non-neoplastic stromal cells can be harvested from various types of solid tumors termed as tumor-derived MSCs, and have been demonstrated to play a more potent role in cancer progression than BM-MSCs do (25).

Hitherto, the cellular and molecular mechanisms of MSC transition from BM-MSCs to tumor-derived MSCs have not been clearly elucidated. Accumulating evidences have indicated that cancer cells or their conditioned medium could induce the activation of MSCs to assume a tumor-promoting phenotype (65, 66). Moreover, tumor-educated neutrophils were also reported to induce the transformation of MSCs into cancer-associated fibroblasts (CAFs), which in turn remarkably facilitated the growth and metastasis of gastric cancer (67). However, there are still few studies on the regulating effect of non-tumor cells on the phenotypes and functions of MSCs, although the cross talk between MSCs and non-tumor cells has been identified to play a dominant role in distinct stages of cancer biology.

Among the non-tumor cells within the inflammatory microenvironment, TAMs have recently been proved to be a key regulator involved in the development and development of gastric cancer (68, 69). The dialogue between MSCs and TAMs has been demonstrated to play a critical role in the establishment of tumor cell proliferation, immune escape, and metastasis. In gastric cancer, TAMs were proved to activate MSCs to acquire CAF-like features, resulting in gastric epithelial cell lesions and malignant transformation via EMT-like changes (70). Another study also demonstrated that TAMs could induce the activation and polarization of MSCs, which subsequently contributed to the development and progression of gastric cancer (Figure 1) (15). Given the multiple active states of TAMs, the phenotypes and functions of MSCs are considered to be complex, which may be influenced by both pro-inflammatory and anti-inflammatory cytokines during distinct stages of gastric cancer (71).

Furthermore, the molecular mechanism underling the regulation of TAMs on MSCs’ transition has also drawn much attention at present. It was demonstrated that the signaling molecules in prostate cancer TME regulated the interplay between BM-MSCs and macrophages during their progression toward malignancy (72). Macrophage-derived TGF-β1 emerged as a crucial molecule able to attract MSC recruitment and induce the transdifferentiation of MSCs, which could in turn recruit and polarize monocyte into an M2 phenotype. In addition, miR-155-5p inhibition could promote the transition of bone marrow-derived MSCs into gastric cancer tissue-derived MSC-like cells (GC-MSCs) via NF-κB p65 activation (73). Another study indicated that BM-MSCs activated by macrophages acquired a pro-inflammatory phenotype, which could promote both gastric epithelial cells and gastric cancer cell proliferation and migration in an NF-κB-dependent manner (15). However, the molecular mechanisms for the “education” or “polarization” of MSCs from MSC1 to MSC2 by TAMs still urgently need to be thoroughly elucidated, since the conveyance of tumor-derived MSC generation will provide new insights into the therapeutic targets for the immunotherapy in gastric cancer.

## The Contributions of Interactions Between MSCs and Tams to Gastric Cancer

### EMT Process and Metastasis

**FIGURE 2:**
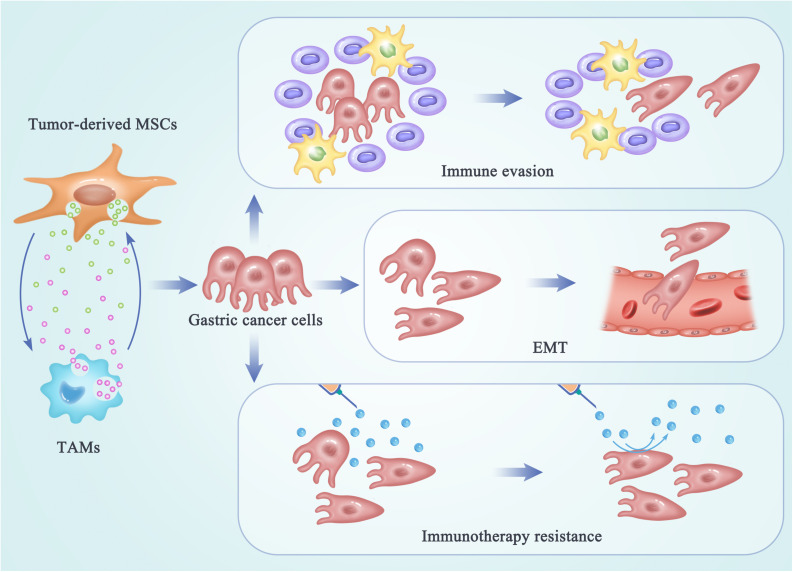
The regulating effects of MSC-TAM interaction on the fate of gastric cancer cells. In the TME of gastric cancer, cross talk between MSCs and TAMs provides a pro-tumor microenvironment by promoting the EMT process and metastasis of gastric cancer cells, as well as the immune escape and immunotherapy resistance in gastric cancer treatments.

Cancer cells undergoing EMT have been demonstrated to be more aggressive, to enhance migratory and invasive properties, and to increase stem-like features and resistance to apoptosis. Therefore, EMT can drive metastasis, drug resistance, and tumor recurrence in the context of cancer. The process of EMT has also been considered as a novel target for immunotherapy of gastric cancer. After interactions between MSCs and TAMs, these two switched tumor-supporting cells may promote tumor progression, metastasis, and drug resistance by enhancing the EMT process in gastric cancer cells (Figure 2). A study reported that MSC-polarized macrophages strikingly promoted the metastasis of gastric cancer by inducing the process of EMT in gastric cancer cells (60). Another research demonstrated that EVs from hypoxia-pre-challenged MSCs can promote the growth and mobility of cancer cells as well as macrophage polarization via miR-21-5p delivery (62). However, molecular mechanisms underlying the regulating effects of MSC-TAM interactions on the EMT progress and metastasis of gastric cancer cells are still not be clarified.

### Immune Evasion

Besides invasion and metastasis, the escape of cancer cells from the immune system is another key limitation leading to high mortality and recurrence in tumor patients. Initially, immune surveillance is able to monitor, recognize, and eliminate the nascent tumor cells. As tumor progresses, cancer cells develop multiple mechanisms of immune evasion, which eventually leads to clinical manifestation of cancer. Recently, the cross talk between non-tumor cell MSCs and TAMs has been reported to be responsible for the formation of a highly immunosuppressive microenvironment, in order to prevent the effective antitumor immune response in the host (Figure 2) (74, 75). Exosomes produced by MSCs were demonstrated to drive differentiation of myeloid cells into highly immunosuppressive M2-polarized macrophages at tumor beds, which could accelerate tumor growth by dampening antitumor immunity (47). Mediated by CD54, the interactions between pro-inflammatory macrophages and MSCs were shown to significantly increase the immunosuppressive capacity of MSCs (76). Reconditioning of the tumor microenvironment and restoration of the competent immune response will be essential for achieving an optimal efficacy of gastric cancer immunotherapy. Therefore, the networks between MSCs and TAMs may provide new targets for attenuating tumor-induced immune tolerance and improving the clinical outcomes in gastric cancer patients.

### Role in Immunotherapy Resistance

Malignant cancer can be considered as a disease of immunological dysfunction. In the TME, interactions between cancer cells and the host’s immune cells determine the elimination or progression of tumors (77). Cancer immunotherapy can induce long-lasting responses and unprecedented tumor regression in the patients with advanced cancer (78). Antibodies that block the immune checkpoints cytotoxic T lymphocyte-associated protein 4 (CTLA-4) and programmed death 1 (PD-1) have been demonstrated to be active in a variety of solid tumors. However, a large portion of patients still do not benefit from the immunotherapy and a fraction of responder relapse, which are due to the immunotherapy resistance (79). Understanding of the cellular and molecular mechanisms underlying immunotherapy resistance may benefit for elucidation of the complex tumor–TME interactions. Reprogrammed by the TME, TAMs can play a tumor-supportive role by limiting the efficacy of immunotherapeutic approaches (80, 81). TAMs may also reprogram MSCs to a state optimal for immunotherapy resistance in tumor niche. However, the molecular and cellular mechanisms underlying the interactions between MSCs and TAMs responsible for the immunotherapy resistance in gastric cancer have not been thoroughly clarified. To improve the outcomes and long-term patient survival, identifying and targeting the complex networks within the TME that facilitate the interactions between MSCs and TAMs will play a potential role in improving the immunotherapeutic efficacy in the future (Figure 2).

## Conclusion

Recent advances in understanding the cellular and molecular mechanisms of tumor pathology have brought us a hope in improving the outcomes in patients with gastric cancer. However, novel targets within the networks of non-tumor cells are still urgently needed for enhancing the clinical efficacy of gastric cancer treatments. In the gastric cancer microenvironment, MSCs and TAMs are two major components with plasticity among the various types of non-tumor cells. In this review, we have elaborated the distinct phenotypic and functional characteristics of either MSCs or macrophages and the interplay of MSCs and macrophages during the development and progression of gastric cancer. By cell-to-cell contact, paracrine effect, or EV transfer, MSCs can affect the polarization of TAMs to obtain an M2-like phenotype. In turn, M2 TAMs trigger the transition of naïve MSCs into tumor-derived MSCs, which may subsequently play a potent role in gastric cancer biology. In addition, the cross talk between MSCs and TAMs might play an essential role in promoting the EMT process and metastasis of gastric cancer cells, as well as immune evasion and immunotherapeutic resistance, which could promptly enhance the development and progression of gastric cancer. Taken together, MSCs and TAMs can be “educated” by each other and present distinct characterizations and functions at tumor beds. Interactions between MSCs or TAMs are complex and active through the initial and developing stages of gastric cancer, which may provide novel and promising targets for immunotherapy in the clinical practice of gastric cancer.

## Perspectives

MSCs and TAMs are both important cells resident within the tumor stroma. Their interactions are associated with the initial and progression of various solid tumors and deserved to be focused on and thoroughly investigated in order to improve the treatment and prognosis of tumor patients. After being “educated” in the TME, either MSCs or TAMs present distinct phenotypes and functions during the development and progression of tumor. However, the cellular and molecular mechanisms underlying the interactions between MSCs and TAMs are still unclear, particularly in gastric cancer. In addition, the transition of MSCs into tumor-derived MSCs by TAMs has not yet been thoroughly investigated, which may provide more promising targets for gastric cancer treatments.

## Author Contributions

PZ wrote the manuscript and drew the figures. WL consulted and revised the manuscript. Both authors read and approved the final manuscript.

## Conflict of Interest

The authors declare that the research was conducted in the absence of any commercial or financial relationships that could be construed as a potential conflict of interest.
